# Age-dependent Powassan virus lethality is linked to glial cell activation and divergent neuroinflammatory cytokine responses in a murine model

**DOI:** 10.1128/jvi.00560-24

**Published:** 2024-08-01

**Authors:** Megan C. Mladinich, Grace E. Himmler, Jonas N. Conde, Elena E. Gorbunova, William R. Schutt, Shayan Sarkar, Styliani-Anna E. Tsirka, Hwan Keun Kim, Erich R. Mackow

**Affiliations:** 1 Department of Microbiology and Immunology, Center for Infectious Disease, Stony Brook, New York, USA; 2 Department of Pathology, Renaissance School of Medicine, Stony Brook University, Stony Brook, New York, USA; 3 Department of Pharmacological Sciences, Renaissance School of Medicine, Stony Brook University, Stony Brook, New York, USA; University of North Carolina at Chapel Hill, Chapel Hill, North Carolina, USA

**Keywords:** Powassan virus, flavivirus, age-dependent lethality, spongiform encephalitis, murine model, neuroinvasion, microgliosis, neurovirulence, neuroinflammation, CNS cytokine responses

## Abstract

Powassan virus (POWV) is an emergent tick-borne flavivirus that causes fatal encephalitis in the elderly and long-term neurologic sequelae in survivors. How age contributes to severe POWV encephalitis remains an enigma, and no animal models have assessed age-dependent POWV neuropathology. Inoculating C57BL/6 mice with a POWV strain (LI9) currently circulating in *Ixodes* ticks resulted in age-dependent POWV lethality 10–20 dpi. POWV infection of 50-week-old mice was 82% fatal with lethality sequentially reduced by age to 7.1% in 10-week-old mice. POWV LI9 was neuroinvasive in mice of all ages, causing acute spongiform CNS pathology and reactive gliosis 5–15 dpi that persisted in survivors 30 dpi. High CNS viral loads were found in all mice 10 dpi. However, by 15 dpi, viral loads decreased by 2–4 logs in 10- to 40-week-old mice, while remaining at high levels in 50-week-old mice. Age-dependent differences in CNS viral loads 15 dpi occurred concomitantly with striking changes in CNS cytokine responses. In the CNS of 50-week-old mice, POWV induced Th1-type cytokines (IFNγ, IL-2, IL-12, IL-4, TNFα, IL-6), suggesting a neurodegenerative pro-inflammatory M1 microglial program. By contrast, in 10-week-old mice, POWV-induced Th2-type cytokines (IL-10, TGFβ, IL-4) were consistent with a neuroprotective M2 microglial phenotype. These findings correlate age-dependent CNS cytokine responses and viral loads with POWV lethality and suggest potential neuroinflammatory therapeutic targets. Our results establish the age-dependent lethality of POWV in a murine model that mirrors human POWV severity and long-term CNS pathology in the elderly.

**IMPORTANCE:**

Powassan virus is an emerging tick-borne flavivirus causing lethal encephalitis in aged individuals. We reveal an age-dependent POWV murine model that mirrors human POWV encephalitis and long-term CNS damage in the elderly. We found that POWV is neuroinvasive and directs reactive gliosis in all age mice, but at acute stages selectively induces pro-inflammatory Th1 cytokine responses in 50-week-old mice and neuroprotective Th2 cytokine responses in 10-week-old mice. Our findings associate CNS viral loads and divergent cytokine responses with age-dependent POWV lethality and survival outcomes. Responses of young mice suggest potential therapeutic targets and approaches for preventing severe POWV encephalitis that may be broadly applicable to other neurodegenerative diseases. Our age-dependent murine POWV model permits analysis of vaccines that prevent POWV lethality, and therapeutics that resolve severe POWV encephalitis.

## INTRODUCTION

Flaviviruses (FVs) are a family of enveloped, positive-strand RNA viruses that cause human disease, and numerous FVs are transmitted by arthropod vectors (1). Tick-borne FVs cause ~15,000 annual cases of severe encephalitis and include the tick-borne encephalitis virus (TBEV) in Eurasia and the Powassan virus (POWV) in North America (2). POWV (strain LB) was first isolated in 1958 from a human encephalitis case in Powassan Ontario, Canada (3). POWVs are emerging in the Northeastern United States due to the expansion of animal reservoirs, tick vectors, and increasing clinical awareness of human POWV infections (2, 4–13). In endemic US states, the seroprevalence of POWV is between 0.7% and 6.1%, with fewer clinical cases suggesting that infections are largely asymptomatic (2, 14). POWV is present in tick saliva, transmitted in as little as 15 minutes of a tick bite (2, 11, 15), and severe cases cause 10% fatal encephalitis and long-term neurologic sequelae in 50% of survivors (2, 16). POWV infects all age groups, with limited data suggesting that lethality and severe neurologic sequelae are increased in patients >60 years of age, similar to clinical outcomes of TBEV infection (2, 13, 17, 18). POWV infections are biphasic with an initial acute febrile illness 1–3 weeks after a tick bite followed by weeks of wide-ranging CNS manifestations (2, 16, 17). Fatal human POWV encephalitis presents with severe CNS damage, brainstem involvement, inflammation, and gliosis in the cerebral cortex, without histologic evidence of systemic infection (2, 16, 17). Currently, there are no clinically approved POWV therapeutics or vaccines.

Animal models are critical for analyzing vaccines and therapeutics and resolving mechanisms of viral neuropathogenesis. There are two POWV genotypes (Lineage I and II) (19, 20); however, POWV strains contain 96% identical Envelope (E) proteins that comprise a single serotype (21, 22) and suggest the broad efficacy of POWV vaccines (23). Many prior POWV murine infection models utilized neuroadapted POWV strains (LB, SP, IPS1) first isolated and serially passaged in murine brains (2, 3, 10, 12, 22, 24–29). Lethality in young 6- to 14-week-old mice is reported to vary by strain, inoculation titers and site, the role of tick salivary glands, and the timing of POWV neuroinvasion (10, 24, 25, 27–32). Infection of 5- to 14-week-old mice with POWV LB results in rapid murine lethality (>60%) 6–9 dpi, while infection with POWVs SP or IPS1 results in delayed and varied mortality (2, 12, 27, 28). Aside from CNS involvement, there is little understanding of POWV-directed encephalitis or mechanisms of POWV lethality in mice (27, 29, 33–36).

TBEV encephalitis is characterized by neuronal damage and glial cell involvement (2, 37–39). CNS pathogenesis includes vacuolation of the neuropil, widespread inflammatory CNS infiltrates, sparse infected Purkinje cells, and dispersed large neurons in the medulla, pons, cerebellum, and striatum (39, 40). However, in a third of human cases, TBEV antigen was undetectable in the CNS (39, 40). In a large TBEV case study, infection was reported in all age groups, with no fatal cases in patients <40 years of age, and increasing fatality rates in patients 60–90 years old (2, 37–39).

In comparison to TBEV, the CDC reported that 8% of POWV patients were <18 years old, and ~50% of patients were >60 years old (2). In a cohort of 99 POWV cases, the median age was 62 years and all fatalities (11%) occurred in patients older than 50 years of age (7). Among 14 cases of POWV encephalitis in New York, 72% of patients were >60 years old, with five fatalities in patients > 60 years of age, and neurologic deficits in all survivors (14, 17). Autopsies revealed reactive gliosis, increased microglia, and necrotizing CNS inflammation consistent with acute meningoencephalitis (2, 14). These findings suggest age as a factor in the severity of encephalitis caused by TBEV and POWV.

In a 2020 survey, 2% of *I. scapularis* ticks in Long Island, NY were POWV positive, and POWV strain LI9 was isolated in Vero cells directly from *Ixodes* ticks, without murine neuroadaptation (21, 41). POWV LI9 non-lytically infects VeroE6 cells, spreading cell-to-cell in the presence of neutralizing antibodies (21, 42), and *in vitro*, LI9 infects primary human brain microvascular endothelial cells (hBMECs) that form the blood-brain barrier (21). Basolateral spread from hBMECs suggests a mechanism for POWVs to enter neuronal compartments (21); however, POWV CNS entry mechanisms have not been assessed *in vivo*. Immunocompetent C57BL/6 mice footpad inoculated with POWV LI9 seroconvert, producing cross-reactive POWV-neutralizing antibodies (21, 42).

Here, we evaluated POWV LI9 neurovirulence and lethality as a function of age across 10- to 50-week-old C57BL/6 mice. Footpad inoculation of POWV LI9 resulted in 82% lethality in 50-week-old mice 10–20 dpi, with lethality reduced sequentially by age to 7.1% in 10-week-old mice. Despite age-dependent differences in lethality, POWV was neuroinvasive in all mice, causing spongiform CNS pathology and reactive gliosis throughout LI9-infected brains 5–15 dpi and in survivors 30 dpi (43–49). CNS viral loads reached similar high levels in all mice 10 dpi, with 2–4 log reductions in 10- to 40-week-old mice by 15 dpi. However, clearance of POWV from the CNS 15 dpi was not observed in 50-week-old mice.

A comparison of CNS responses to POWV 15 dpi revealed the robust induction of pro-inflammatory Th1-type cytokines (IL-2, IL-6, IL-12, IL-1β, TNFα, and IFNγ) in 50-week-old mice, and discrete Th2-type cytokines (IL-10, TGF-β, and IL-4) and chemokines in 10-week-old mice. These findings are consistent with a neurodegenerative M1 microglial phenotype (46, 48, 50–59) in 50-week-old mice and a neuroprotective M2 microglial phenotype (53, 57, 60, 61) in 10-week-old mice. Collectively, these findings correlate age-dependent differences in CNS viral clearance and glial cell activation phenotypes with POWV lethality (43, 48, 58, 59, 62, 63). Studies establish a murine model of age-dependent POWV lethality that reflects the severity of POWV disease and long-term neurodegenerative pathology seen in elderly patients (2, 7, 13, 14).

## RESULTS

### POWV LI9 lethality is age-dependent in C57BL/6 mice

**Fig 1:**
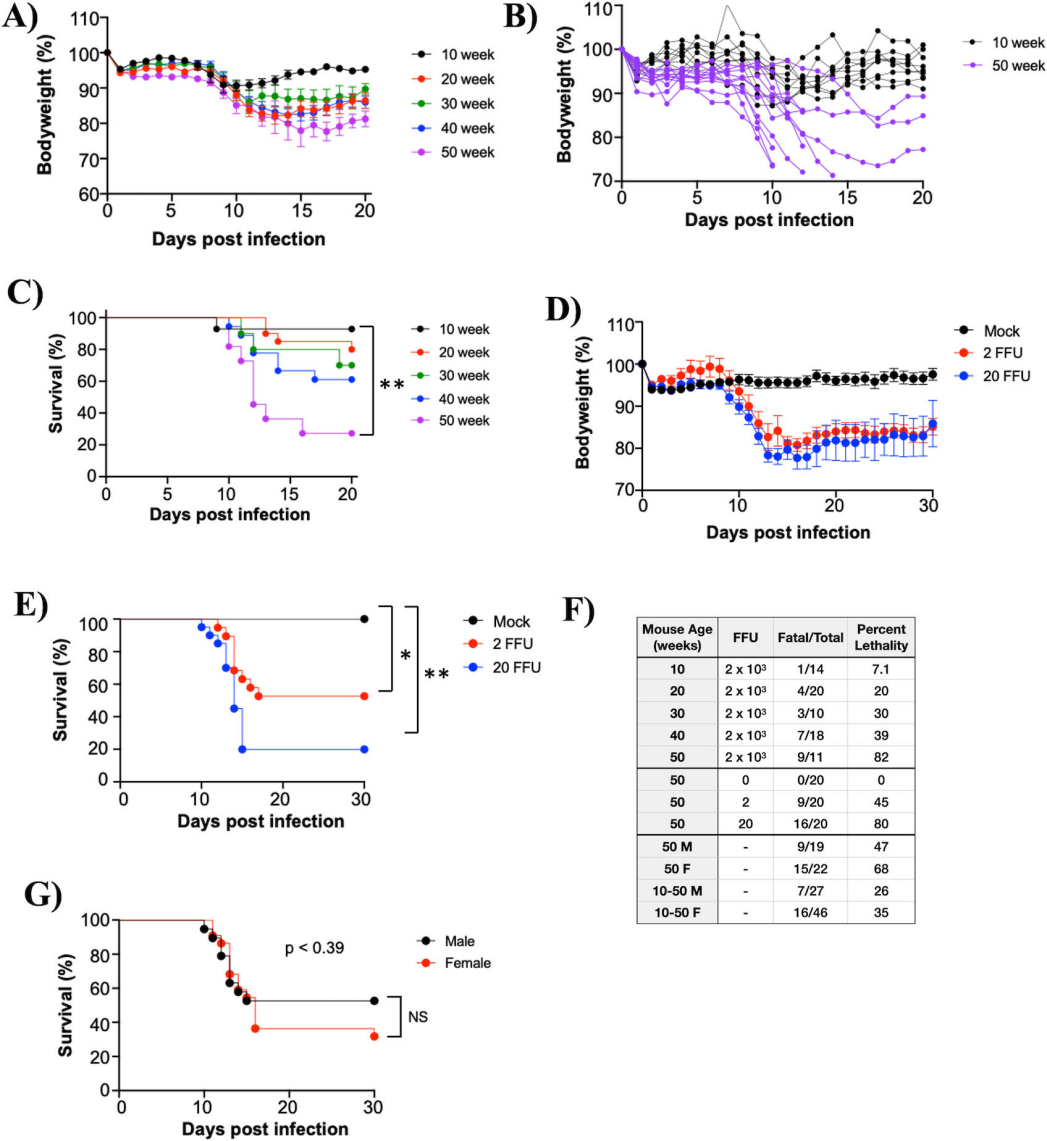
The lethality of POWV LI9 is age-dependent in C57BL/6 mice. (**A–G**) 10- to 50-week-old C57BL/6 mice were footpad inoculated with 2 × 10^3^ FFU (**A, B, C, G**), or 2–20 FFU (**D, E**) of POWV LI9 or PBS. (**A, B, D**) Mice were weighed daily, and the appearance of clinical neurologic signs (hindlimb flaccid paralysis, ataxia, weak grip, and failure to right) was used to determine humane lethal endpoints (64). (**A**) Body weights of individual 10- to 50-week-old mice are presented as a percent of their original weights prior to inoculation (mean ± standard error of the indicated number of mice per group). (**B**) Body weight changes for each individual 10-week and 50-week-old mouse are comparatively presented. (**C, E**) Kaplan-Meier curves of lethal POWV infection of 10- to 50-week old mice (**C**): 10-week (*n* = 14), 20-week (*n* = 20), 30-week (*n* = 10), 40-week (*n* = 18), 50-week (*n* = 11) -old mice; and (**E**) 50-week-old mice inoculated with 2 FFU (*n* = 20), 20 FFU (*n* = 20), or mock infected (*n* = 20) were analyzed by a log-rank test (*P < 0.05; **P < 0.01). (**F**) Summary of murine age, POWV LI9 inoculation dose, fatal/total infected, and percent lethality. Overall lethality of POWV in male (*n* = 27) vs female (*n* = 46) mice across all ages was not significant (NS; *P* = 0.41). (**G**) Kaplan-Meier curve of lethal POWV infection of 50-week-old male (*n* = 19) vs 50-week-old female (*n* = 22) mice inoculated with 2 × 10^3^ FFU, analyzed by a log-rank test, was not significant (NS; *P* = 0.39). (**A–G**) Data are cumulative from three independent experiments.

POWV LI9 is a currently circulating virus isolated directly from *Ixodes* ticks in VeroE6 cells (21). Tick-transmitted TBEV and POWV are associated with the age-dependent severity of human encephalitis (2, 7, 14, 17, 37–39). To investigate age as a determinant of LI9 lethality, we footpad inoculated 10-, 20-, 30-, 40-, and 50-week-old C57BL/6 mice with LI9 (2 × 10^3^ FFU) and assessed lethal neurovirulent disease (Fig. 1). In all age groups, LI9 infection resulted in clinical neurologic signs including hindlimb flaccid paralysis, ataxia, weak grip, failure to right, and weight loss (Fig. 1A and B) (64). POWV LI9 lethality was determined by clinical neurologic signs observed at 10–20 dpi. LI9 infection caused a sequential age-dependent increase in lethality from 7.1% (10 weeks), 20% (20 weeks), 30% (30 weeks), 39% (40 weeks) to 82% (50 weeks), (*n* = 11–20/group; Fig. 1C and F). All surviving mice seroconverted and produced neutralizing POWV antibodies as determined by focus reduction neutralization assay (IC50 range: 7 × 10^2^ to 1 × 10^4^, Table 1). Footpad inoculation of 50-week-old mice with 2 or 20 FFUs of LI9 (*n* = 20/group) resulted in weight loss (Fig. 1D) and neurologic signs resulting in 45% and 80% lethality in LI9 infected mice, respectively (Fig. 1E and F). The mortality of LI9-infected male versus female mice, summed across all ages, or exclusively in 50-week-old mice, was not statistically significant (Fig. 1F and G). Collectively, the data reveal that minimal infectious doses of POWV LI9 (2–20 FFUs) are highly lethal in 50-week-old mice and that POWV LI9 lethality increases with the age of C57BL/6 mice.

**TABLE 1:** Neutralizing antibody titers of 10- to 50-week old mice following POWV LI9 infection

Mouse age (weeks)	Neutralizing Ab IC50 (30 dpi)
10	1.0 × 10^3^–2.0 × 10^3^
20	1.2 × 10^3^–2.7 × 10^3^
30	9.8 × 10^2^–2.4 × 10^3^
40	6.8 × 10^2^–1.6 × 10^3^
50	4.6 × 10^3^–1.2 × 10^4^

### POWV causes spongiform encephalitis and CNS resident glial cell activation

**Fig 2:**
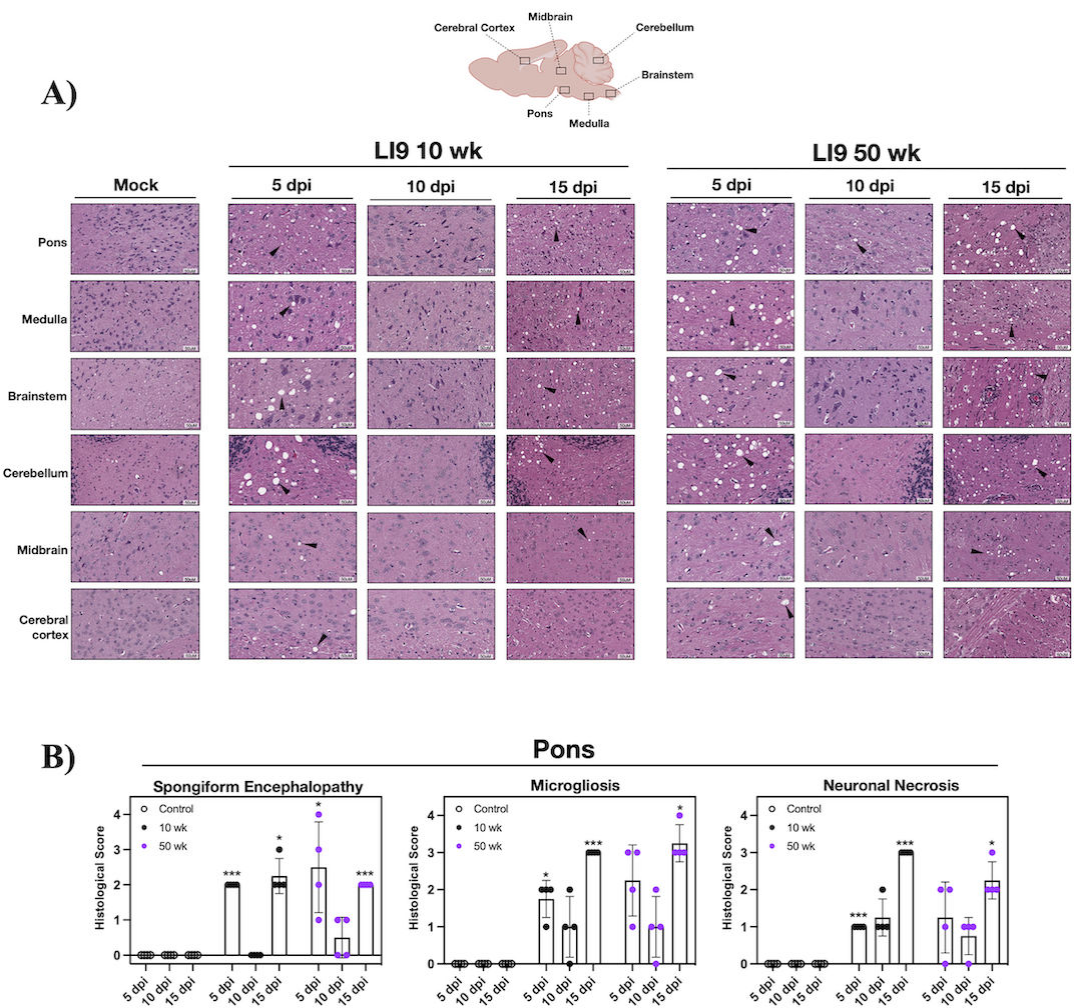
POWV causes spongiform encephalitis and microgliosis in mice. (**A and B**) C57BL/6 10- and 50-week-old mice were footpad inoculated with 2 × 10^3^ FFU of POWV LI9 or mock infected with PBS. Brains were harvested 5, 10, and 15 dpi, and H&E stained (*n* = 4). (**A**) Representative images of CNS histopathology in the pons, medulla, brainstem, cerebellum, midbrain, and cerebral cortex of POWV versus mock-infected 50-week-old brains are presented. (**B**) The severity of POWV-directed spongiform encephalopathy, microgliosis, and neuronal necrosis in H&E-stained brains (*n* = 4) was scored on a scale of 0–4 by blinded comparison versus age-matched controls: (0) baseline determined by control brain staining in select region, (1) localized lesion, (2) multiple localized lesions, (3) lesions spread throughout most of selected region, (4) lesions uniformly spread throughout select region (71). Scores of the pons are presented. Data are presented as means with SD and each dot represents an individual mouse. Individual data point comparisons were performed by two-way ANOVA analysis. Asterisks indicate statistical significance (*P < 0.01; **P < 0.001, ***P < 0.0001).

**Fig 3:**
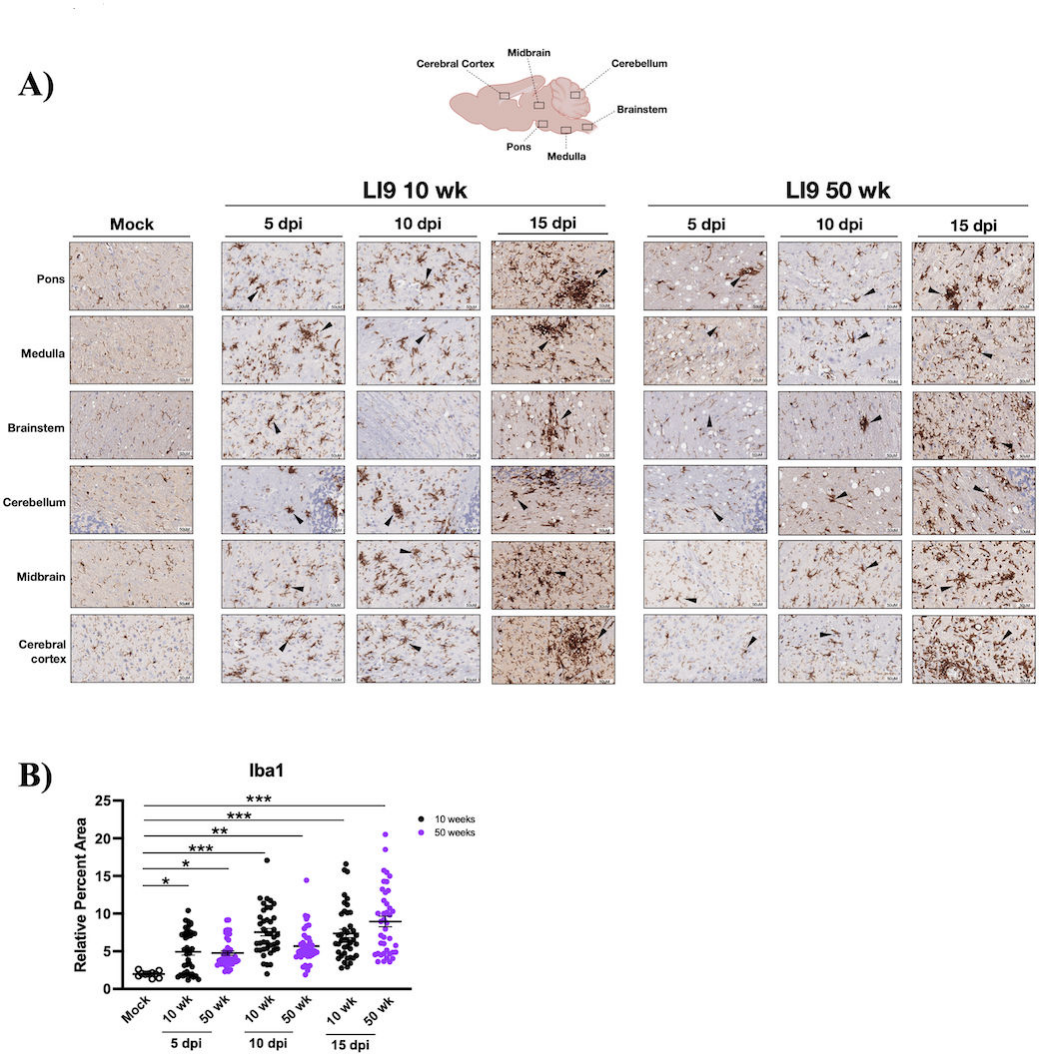
POWV infection causes IBA1^+^ microgliosis in mice. (**A, B**) 10- and 50-week-old C57BL/6 mice were footpad inoculated with 2 × 10^3^ FFU of POWV LI9 or mock infected with PBS. Brains were harvested 5, 10, and 15 dpi, sectioned, and immunostained for microglial/macrophage markers using anti-Iba1 antibody (*n* = 4). (**A**) Representative images of CNS histopathology in the pons, medulla, brainstem, cerebellum, midbrain, and cerebral cortex of POWV versus mock-infected 50-week-old brains are presented. Arrows indicate representative Iba1^+^ microglial nodules. (**B**) ImageJ quantification of Iba1 immunostaining: relative percent area of Iba1^+^ pixel intensity was determined for 10 CNS regions of 10- and 50-week-old mice (*n* = 4), 5, 10, and 15 dpi, vs mock-infected controls. Data are presented as means with SEM and each dot represents an individual mouse. Individual data point comparisons were performed by two-way ANOVA analysis. Asterisks indicate statistical significance (*P < 0.01; **P < 0.001, ***P < 0.0001).

**Fig 4:**
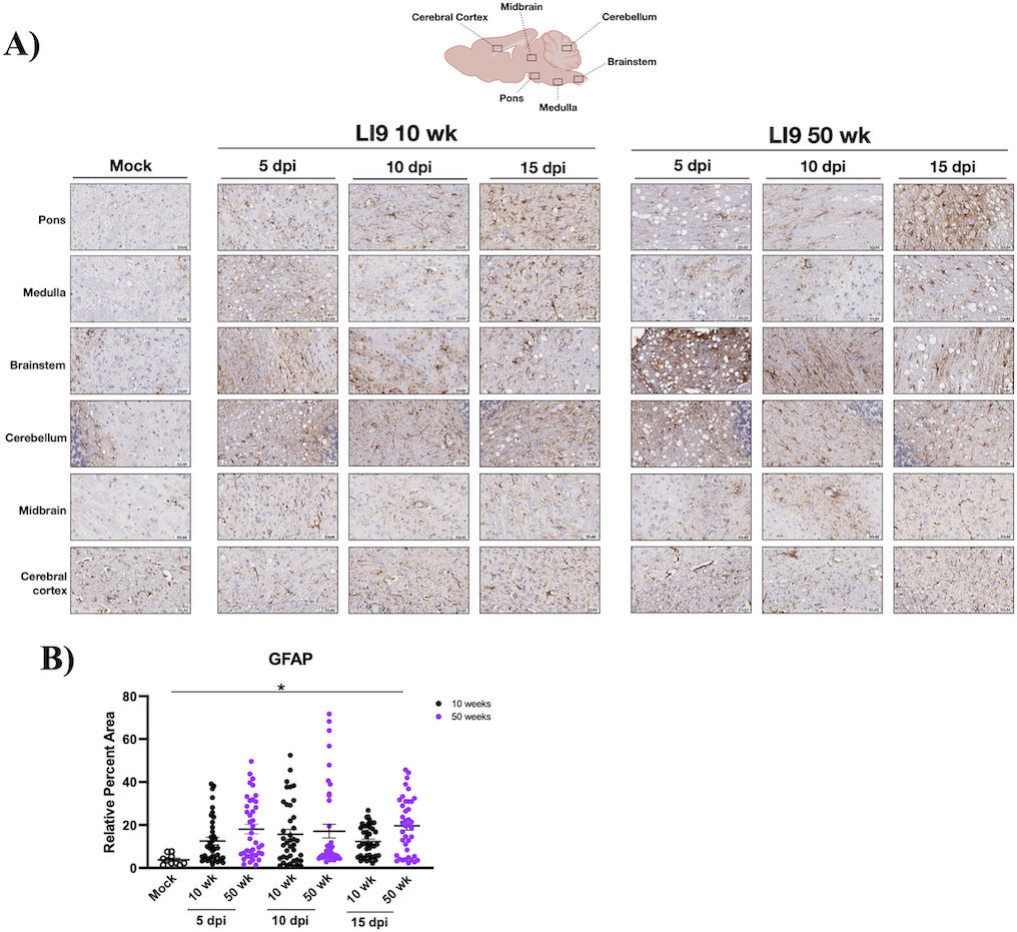
POWV infection causes astrocyte activation in mice. (**A and B**) C57BL/6 10- and 50-week-old mice were footpad inoculated with 2 × 10^3^ FFU of POWV LI9 or mock infected with PBS. Brains were harvested 5, 10, and 15 dpi, sectioned, and anti-GFAP immunostained to identify activated astrocytes. (**A**) Representative images of astrocytosis in the pons, medulla, brainstem, cerebellum, midbrain, and cerebral cortex of POWV- versus mock-infected 50-week-old brains are presented. (**B**) ImageJ quantification of GFAP immunostaining: relative percent area of GFAP^+^ pixel intensity was determined for 10 CNS regions of 10- and 50-week-old mice (*n* = 4), 5, 10, and 15 dpi, vs mock-infected controls. Data are presented as means with SEM and individual data point comparisons to mock-infected controls were performed by two-way ANOVA analysis. Asterisks indicate statistical significance (*P < 0.05).

**Fig 5:**
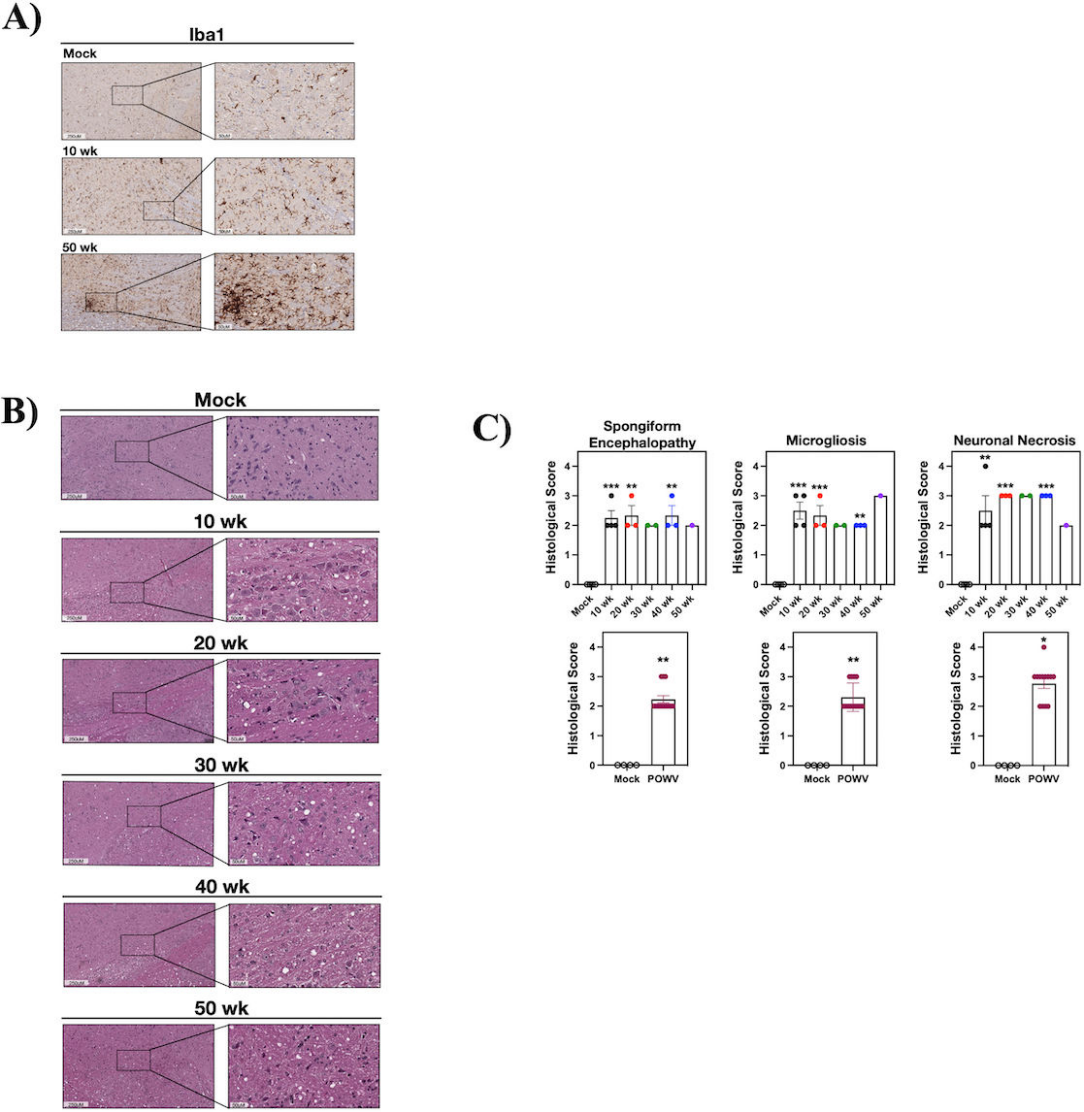
POWV causes persistent spongiform encephalopathy and microgliosis 30 dpi. (**A–C**) C57BL/6 mice (10-, 20-, 30-, 40-, and 50-week-old mice) that survived infection with 2 × 10^3^ FFU POWV LI9 were euthanized 30 dpi. Brains were harvested, sectioned and either (**A**) anti-Iba1 immunostained or (**B**) H&E stained. (**A**) Representative images of the Iba1^+^ immunostained pons of POWV-infected 10- and 50-week-old mice 30 dpi and mock-infected 50-week-old mice. (**B**) Representative images of CNS histopathology in the pons of 10-, 20-, 30-, 40-, and 50-week-old POWV-infected mice at 30 dpi versus mock-infected 50-week-old brains are presented. (**C**) The severity of POWV-directed spongiform lesions, microgliosis and neuronal necrosis 30 dpi was scored on a scale of 0–4 versus age-matched mock-infected 50-week-old control as described in Fig. 2B. Data are presented as means with SD and each dot represents an individual mouse (Control *n* = 4; 10 weeks *n* = 4; 20 weeks *n* = 3; 30 weeks *n* = 2; 40 week *n* = 3; 50 weeks *n* = 1). Individual data point comparisons were performed by one-way ANOVA on age groups with *n* = 3–4 mice. The severity of spongiform encephalopathy, microgliosis, and neuronal necrosis of all POWV-infected (*n* = 13) mice 30 dpi were compared to uninfected 50-week-old controls (*n* = 4) and analyzed by student’s *t*-test. Asterisks indicate statistical significance (*P < 0.05; **P < 0.01, ***P < 0.001).

CNS histopathology following POWV LI9 footpad infection of 10- to 50-week-old mice was evaluated on formalin-fixed brains 5, 10, 15, and 30 dpi. Astrocytes and microglia comprise CNS resident glial cells, and reactive gliosis is an early marker of CNS damage (65). Glial cells direct immune surveillance, amplify neuroinflammatory cytokine responses, and orchestrate discrete neurodegenerative and neuroprotective CNS responses (44, 47–49, 58, 59, 62, 63). Representative sections from six discrete brain regions (*n* = 4/ time point and age) were H&E stained (Fig. 2A), anti-Iba1 immunostained for reactive microglia/macrophages (Fig. 3A), and anti-GFAP (glial fibrillary acidic protein) immunostained for reactive astrocytes (Fig. 4A) (48, 66). We observed striking spongiform encephalopathy with severe neuronal necrosis, glial scarring, and perivascular cuffing in the pons, medulla, brainstem, and cerebellum compared to age-matched controls (Fig. 2 to 4) (67). H&E staining revealed rapid spongiform vacuolation 5 dpi in the CNS of all POWV-infected mice (Fig. 2; Fig. S1) that occurred concomitantly with increased neuronal necrosis, reactive microgliosis (Fig. 2B; Fig. S1B), and reduced NeuN immunostaining of neurons 5 dpi (Fig. S2A). In all POWV-infected mice, a reduction in spongiform vacuoles was observed at 10 dpi, consistent with the timing of proliferative glial cell repair normally observed following acute CNS damage (68–70). This was followed by an increase in spongiform vacuoles throughout the CNS 15–30 dpi (Fig. 2 and 5; Fig. S1). Scoring sections of the pons 5–15 dpi for spongiform encephalopathy, microgliosis, and neuronal necrosis (*n* = 4) (71) revealed similar pathology in 10- to 50-week-old LI9-infected mice versus age-matched controls (Fig. 2; Fig. S1 and S3).

Purkinje cells are reported targets of TBEV, POWV LB, and West Nile virus (WNV) encephalitis (29, 36, 39, 72, 73), and noted at autopsy of immunosuppressed POWV patients (16, 74). Despite the early vacuolization and loss of neurons (NeuN immunostained) in the LI9-infected CNS (Fig. S2A), we found no histologic evidence of infection, depletion or damage to granular, Purkinje or molecular layers of the cerebellum in POWV-infected 10- or 50-week-old mice 5–30 dpi (Fig. S2B).

Increases in inflammatory Iba1^+^ staining, reflective of reactive microglial nodules and microgliosis (75, 76), were apparent in all CNS regions of 10- and 50-week-old mice (Fig. 3A). Iba1^+^ staining of the pons increased from 5 to 15 dpi, as assessed by ImageJ quantification (10 areas per mouse, *n* = 4; Fig. 3B). Increases in reactive glia from 5 to 15 dpi were also observed and quantified in the pons of LI9-infected 20- to 40-week-old mice (Fig. S1 and S3). Similar analysis and quantification revealed increases in GFAP^+^ astrocytes from 5 to 15 dpi in 10- and 50-week-old POWV-infected mice (Fig. 4). These results demonstrate that POWV is neuroinvasive, causing rapid CNS neuronal damage by 5 dpi, with dramatic increases in reactive glial cells from 5 to 15 dpi in the CNS of all age mice.

### CNS damage and microgliosis persist in POWV-infected survivors 30 dpi

POWV causes long-term cognitive deficits in 50% of surviving patients (2, 7, 14). In surviving mice 30 dpi, we observed reactive Iba1^+^ glial cells in the Pons of both 10- and 50-week-old POWV survivors (Fig. 5A). Brains from POWV-infected murine survivors 30 dpi revealed persistent long-term CNS spongiform pathology, microgliosis, and neuronal necrosis, in all age mice (10-, 20-, 30-, 40-, and 50-week-old mice) versus uninfected controls (Fig. 5B). Similar increases in scored spongiform pathology, microgliosis, and neuronal necrosis were observed in the pons of all LI9-infected mice 30 dpi versus 50-week-old controls within age groups, and by comparison of all POWV-infected (*n* = 13) versus -uninfected (*n* = 4) controls (Fig. 5C). These findings indicate that following POWV LI9 infection, neurodegenerative CNS damage persists in the CNS of all surviving mice, and that long-term CNS pathology is age independent.

### POWV-infected CNS contain few CD4 and CD8 T-cell infiltrates

**Fig 6:**
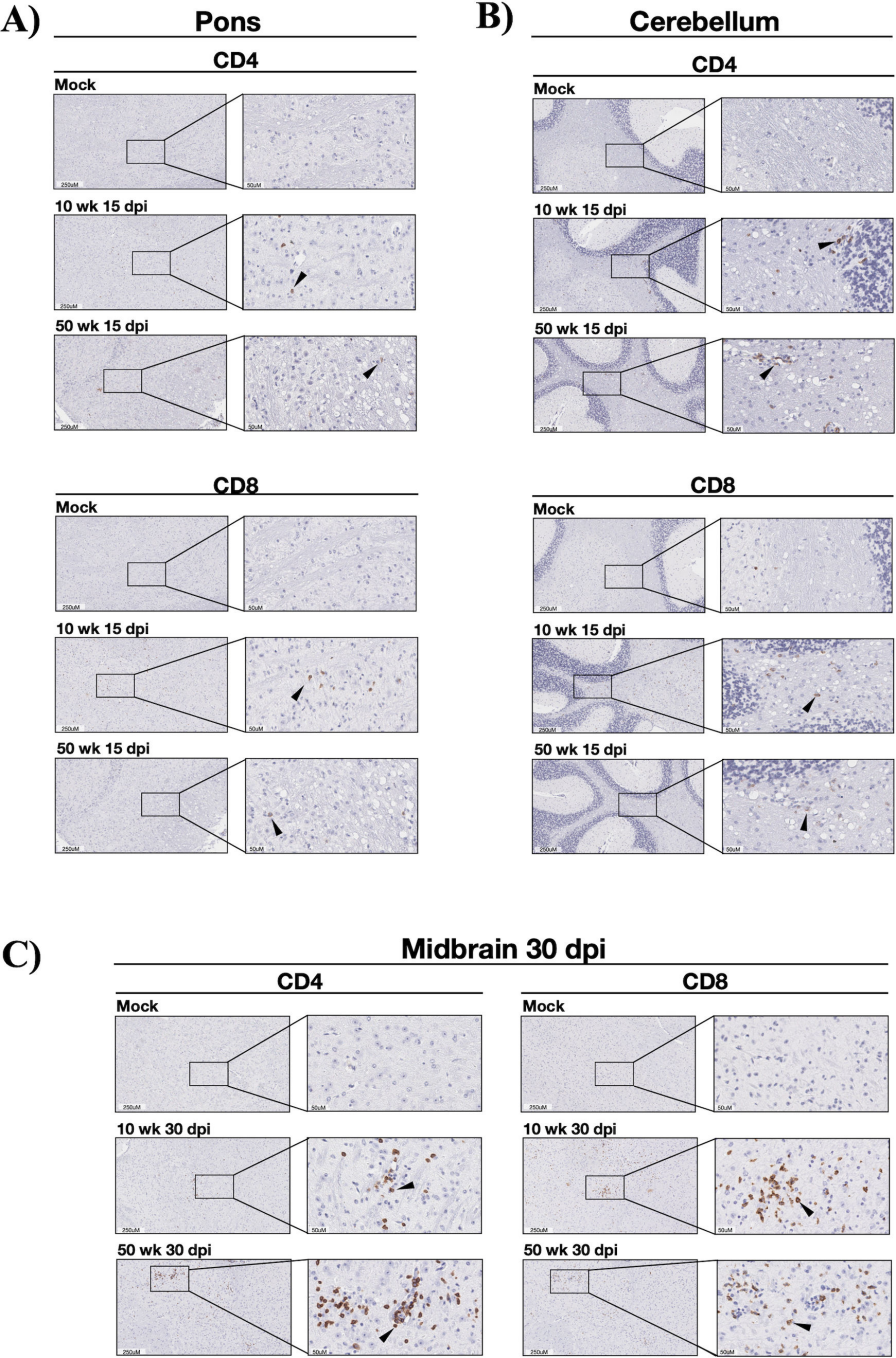
POWV-infected CNS CD4 and CD8 T-cell immunostaining. (**A–C**) C57BL/6 10- and 50-week-old mice were footpad inoculated with 2 × 10^3^ FFU of POWV LI9 or mock infected with PBS. Brains were harvested at 15 dpi and immunostained for T-cell infiltrates using antibodies to CD4 or CD8 (*n* = 4). Arrows indicate representative CD4^+^ or CD8^+^ immunostained cells. (**A**) Representative sections of the pons of POWV-infected 10- and 50-week-old mice 15 dpi versus mock-infected 50-week controls are presented. (**B**) Representative sections of the cerebellum of POWV-infected 10- and 50-week-old mice 15 dpi versus mock-infected 50-week controls are presented. Black arrows indicate representative CD4 or CD8 immunostained cells. (**C**) Representative sections of the midbrain of POWV-infected 10- and 50-week-old mice 30 dpi versus mock-infected 50-week controls are presented.

In the POWV-infected CNS, T-cell infiltrates have yet to be evaluated. Following TBEV infection, only a few CD4^+^ T cells were detected in the CNS, and despite TBEV lethality in CD8^-/-^ C57BL/6 mice, CD8^+^ T cells were suggested to mediate CNS immunopathology (77, 78). We evaluated CD4^+^ and CD8^+^ T-cell infiltrates by immunostaining POWV-infected brain sections. Regardless of overt spongiform damage and glial cell activation in LI9-infected mice 15 dpi, CD4^+^ or CD8^+^ T-cell infiltrates were virtually absent from any area of the CNS of 10- or 50-week-old mice. In acutely infected 10- and 50-week-old mice, only rare infiltrating CD4^+^ T cells were stained in any CNS region, with a few CD8^+^ T cells sparsely dispersed in the pons and cerebellum (Fig. 6A and B). Maximal CD4- and CD8-positive areas of the CNS 15 dpi are presented but lack quantifiable levels (Fig. 6A and B). Thus, CNS CD4^+^ and CD8^+^ T-cell responses 15 dpi differ dramatically from abundant reactive glial cell levels, which suggest a selective role for glial cell responses in acute phases of POWV encephalitis (43, 44, 47, 58, 59, 79–84). In contrast to acute phases of infection, at 30 dpi, higher levels of CD4^+^ and CD8^+^ T cells in distinct foci were observed in the CNS of surviving 10- and 50-week-old mice (Fig. 6C). Consequently, reactive glial cell and CD4/CD8 T-cell responses may both contribute to long-term CNS inflammation in POWV survivors.

### Age-dependent lethality is linked to POWV CNS load

**Fig 7:**
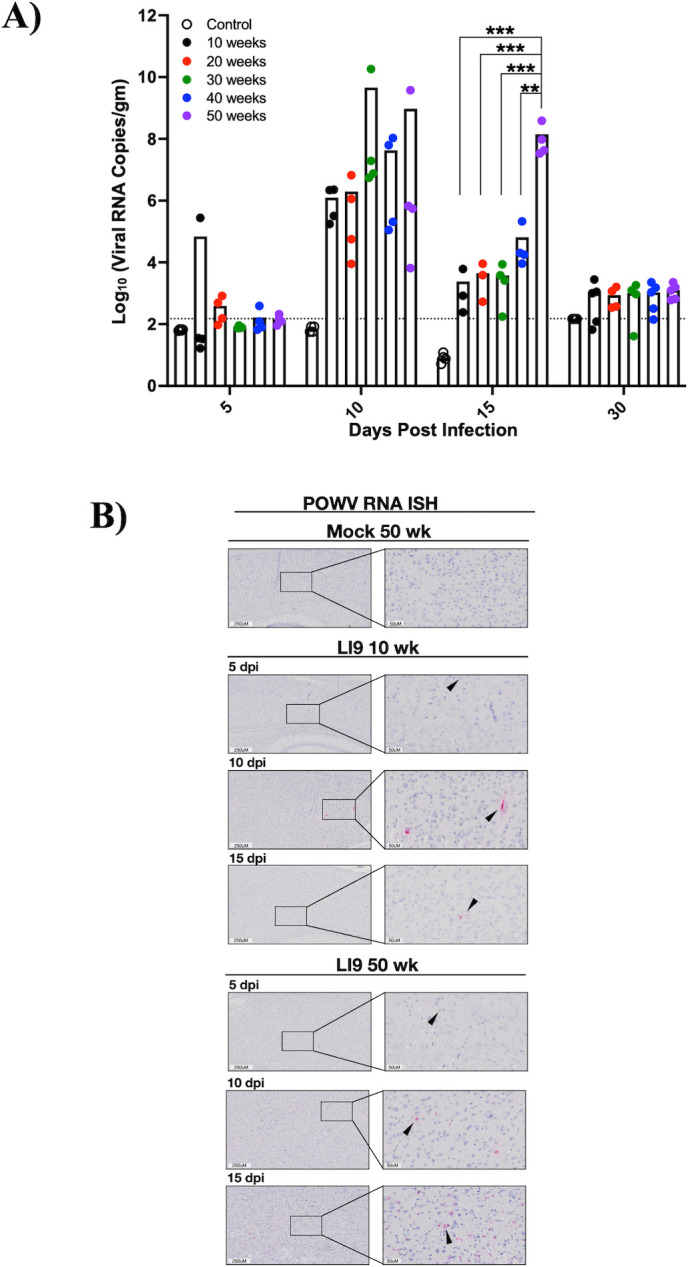
POWV CNS load kinetics and ISH analysis. C57BL/6 10- to 50-week-old mice were footpad inoculated with 2 × 10^3^ FFU of POWV LI9 or PBS. (**A**) POWV RNA levels in the CNS were assayed by qRTPCR 5–30 dpi and compared to mock-infected controls. Data are expressed as the Log_10_ of POWV RNA (copies/gm) normalized to a standard curve. Each time point and age group reflect an *n* = 4 following POWV infection and *n* = 11 for mock-infected controls. Data are presented as means and each dot represents an individual mouse. Individual data point comparisons were performed by two-way ANOVA analysis of log_10_ transformed values. Asterisks indicate statistical significance (**P < 0.001, ***P < 0.0001). (**B**) Brains from POWV-infected or mock-infected 10- and 50-week-old mice were harvested at 5, 10, and 15 dpi, and genomic POWV RNA in the CNS was detected by *in situ* hybridization (ISH) with POWV RNAscope probe-red (ACD). Representative images of POWV RNA ISH in the cerebral cortex of POWV-infected (*n* = 3) and mock-infected 50-week-old mice (*n* = 1) are presented. Black arrows indicate representative cells positive for genomic POWV RNA by ISH.

Viral load in the CNS of LI9-infected 10- to 50-week-old mice was measured at 5, 10, 15, and 30 dpi by qRT-PCR. LI9 RNA was detected in murine brains of all age groups (*n* = 4 per age and dpi). While only 1/20 mice had detectable LI9 RNA levels 5 dpi, by 10 dpi all murine age groups had >10^6^ copies/g of LI9 RNA with the highest CNS viral levels found in 30- to 50-week-old mice 10 dpi (10^8^–10^10^ copies/g, Fig. 7A). However, by 15 dpi, viral RNA in the CNS of 10-, 20-, and 30-week-old mice decreased to <10^4^ copies/g, with RNA levels in 40-week-old mice decreased to ~10^5^ copies/g (Fig. 7A). By contrast, 15 dpi LI9 RNA levels remained at ~10^8^ copies/gm in the brains of 50-week-old mice (Fig. 7A). Mice of all ages that survived lethal infection (30 dpi) had comparable viral loads that were near the level of detection (Fig. 7A). *In situ* hybridization (ISH) of positive-stranded POWV RNA in the CNS (27, 85, 86) failed to detect POWV RNA in the CNS 5 dpi, and detected sporadic POWV RNA in single-cell foci dispersed throughout the brains of 10- and 50-week-old mice, 10–15 dpi (Fig. 7; Fig. S4). ISH staining of POWV RNA was notably increased in the cerebral cortex (Fig. 7B) and midbrain (Fig. S4) of 50-week-old brain sections 15 dpi. Collectively, these findings demonstrate that POWV LI9 neuroinvasion is age independent, with high viral loads in the CNS of all age mice 10 dpi. However, the dramatic reduction in POWV viral load in younger mice from 10 to 15 dpi was not observed in 50-week-old mice, where high CNS viral loads occur coincident with acute infection and 82% lethality.

### POWV directs age-dependent cytokine responses that distinguish CNS microglial phenotypes

**Fig 8:**
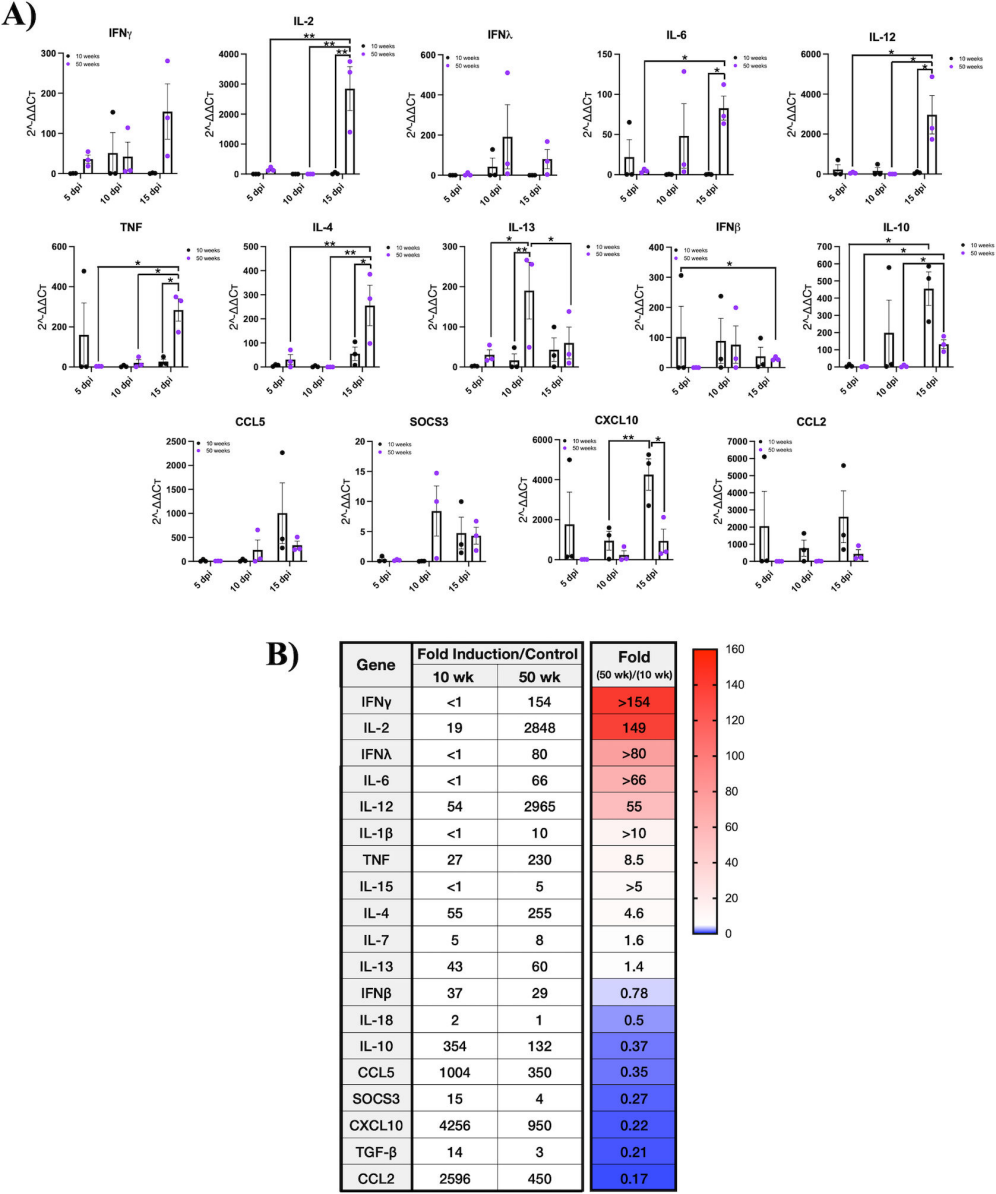
POWV induction of CNS cytokines and chemokines is age dependent. (**A**) C57BL/6 10- and 50-week-old mice were footpad inoculated with 2 × 10^3^ FFU of POWV LI9 or mock infected with PBS. Brains from POWV or mock-infected mice were harvested, total RNA extracted and the induction of cytokine and chemokine transcripts were analyzed kinetically (5, 10, and 15 dpi) for fold induction over age-matched controls by qRT-PCR after standardizing to GAPDH RNA levels. At each time point, age group-infected or mock-infected age-matched control is presented (*n* = 3). Data are presented as means with SEM and each dot represents an individual mouse. Individual data point comparisons were performed by two-way ANOVA analysis. Asterisks indicate statistical significance (*P < 0.05; **P < 0.01). (**B**) A summary of the fold induction of 10- and 50-week-old CNS cytokine and chemokine responses over age-matched controls 15 dpi is presented. Findings are sorted by the fold induction of 50-week/10-week-old CNS responses with fold differences and a heat map that segregates age-dependent responses.

Reactive responses of CNS resident glial cells promote T helper (Th) cell effector functions by directing pro-inflammatory Th1-type cytokines or anti-inflammatory Th2 cytokine responses that in the CNS divergently contribute to neuropathology and neuroprotection (1, 44, 46, 52, 57, 59, 62, 63, 84). Following POWV LI9 infection of 10- and 50-week-old mice, we assayed induced CNS cytokine and chemokine responses 5, 10, and 15 dpi by qRT-PCR, and compared responses to age-matched controls (Fig. 8A). Evaluation of responses revealed consistent high-level induction of IL-2, IL-12, TNFα, and IFNγ at 15 dpi in only 50-week-old mice (Fig. 8; Fig. S5). Further comparison of CNS responses in POWV-infected 10- and 50-week-old mice revealed a 5- to 154-fold induction of pro-inflammatory Th1-type cytokines (IL-2, IL-6, IL-12, IL-15, TNFα, IL-1β, and IFNγ in the CNS of 50-week-old mice over 10-week-old mice 15 dpi (Fig. 8B; Fig. S5) (50–52, 55, 87, 88). By contrast, in the CNS of 10-week-old mice POWV-induced anti-inflammatory Th2-type cytokines IL-10 and TGF-β (53, 57, 59, 61, 63, 89, 90) and CCL2, CCL5, and CXCL10 chemokines, 3- to 5-fold higher than 50-week-old mice (Fig. 8B). IL-4, induced in both 10- and 50-week mice, directs Th2-type responses, but in the context of IL-2 or IL-12, promotes inflammatory Th1-type cytokines (51, 56, 59, 87). CNS responses observed in 50-week-old mice are consistent with inflammatory M1 microglial activation directing neurodegenerative Th1-type cytokines (Fig. 8B, red/white), while in 10-week-old mice, distinct neuroprotective Th2-type cytokine responses (Fig. 8B, blue) are consistent with M2 microglial activation (57, 59, 63, 91, 92). Our findings reveal a potential mechanism of age-dependent POWV lethality resulting from POWV neuroinvasion, sustained high CNS viral load, and the differential induction of late, inflammatory versus neuroprotective, cytokine responses. These results suggest potential therapeutic targets for resolving POWV lethality in a murine model that mirrors POWV disease in the elderly (16).

## DISCUSSION

Severe encephalitis and long-term neurologic damage are associated with human POWV infections (2, 16), but mechanisms of POWV neuropathology and lethality remain largely unknown. Despite a lack of systemic infection or peripheral disease, POWV patients display profound cerebellar and brainstem involvement, characterized by neuronal loss, microgliosis, inflammatory infiltrates, and POWV RNA in the CNS (2, 16). Data available from a limited number of human POWV cases indicates that many lethal POWV infections occur in individuals >60 years of age (2, 7, 14, 17) and are consistent with the lethality of TBEV infections (38, 39) and the age-dependent severity of WNV encephalitis (79, 93–95).

Models of POWV infection were previously studied in peripherally inoculated 5- to 14-week-old mice primarily using murine brain neuroadapted LB and SP strains (2, 12, 24, 25, 27, 29, 30). In these studies, lethality varied by strain, dose, kinetics, and suggested requirements for tick saliva in directing neurovirulence (29, 36). Here, we assessed the age-dependent lethality of POWV in 10- to 50-week-old mice using a LI9 POWV strain present in circulating *Ixodes* ticks that was directly isolated and passaged in VeroE6 cells (21, 42). Footpad inoculation of POWV LI9 into C57BL/6 mice resulted in 82% lethality in 50-week-old mice, with lethality sequentially reduced with age to 7.1% in 10-week-old mice (Fig. 1C and F). Murine lethality was not sex linked, and a minimal infectious dose of LI9 was 80% fatal in 50-week-old mice (Fig. 1E through G). These findings establish the age-dependent lethality of POWV LI9 in a murine model that reflects the severity of human POWV disease in the elderly (13).

Similar to CNS degeneration observed at autopsy of POWV patients, histopathology of LI9-infected mice revealed spongiform CNS lesions in cerebellar and hindbrain regions of the pons, medulla, and brainstem that were accompanied by neuronal depletion and the presence of reactive microglial nodules (2, 13, 16, 65). We analyzed four time points (5, 10, 15, and 30 dpi) post-POWV infection for CNS histopathology, viral load, glial, and cytokine responses across murine age. At 5 dpi, there were no clinical signs of POWV infection, and CNS viral loads were at minimal levels or undetected by qRT-PCR or ISH. Yet, the CNS of all age mice contained focal spongiform lesions, neuronal depletion, and reactive CNS resident glial cells 5 dpi. This suggests that early damage to the CNS following POWV infection is age-independent, but fails to reveal potential causes of POWV lesions (neuroinvasion, apoptosis, glial cell phagocytosis, etc.) or if neurodegenerative or cognitive deficits are common to all POWV survivors (2, 7, 9, 14, 16, 17, 96).

We found high POWV loads in the CNS of all age mice at 10 dpi, before most mice show clinical neurologic signs. Comparably high CNS viral loads 10 dpi indicate that POWV neuroinvasion and replication in the CNS are age-independent and distinct from subsequent late-stage age-dependent lethality. CNS histopathology 10 dpi revealed a marked reduction in spongiform lesions in mice of all ages, and the increased presence of reactive microglia/macrophages and astrocytes (44–49, 58, 59, 62, 75, 76, 91, 97, 98). These findings are consistent with CNS cell proliferation, tissue replacement, and repair that occurs 2–10 days after CNS damage and is directed by reactive glial and neuronal progenitor cells (68–70). Thus, at 10 dpi, glial cell proliferation, inflammation, and tissue repair in the CNS occur simultaneously with high levels of POWV replication, and the consequence of these opposing responses appears pivotal in determining murine survival or lethality.

Despite age-dependent differences in POWV lethality, in all age mice clinical signs and lethality were observed between 10 and 20 dpi (1/49 fatalities occurred 9 dpi). Analysis of mice at an acute lethal stage of infection, 15 dpi, revealed focal spongiform vacuoles in all aged mice with increased levels of reactive glia cells throughout the CNS. At 15 dpi, a reduction in CNS viral loads was noted in 10- to 40-week-old mice, while POWV loads in the CNS of 50-week-old mice remained undiminished and identical to CNS viral loads found in moribund 50-week-old mice. These findings suggest potential age-dependent differences in POWV clearance from the CNS, replication rates, CNS damage (POWV or immune mediated), or discrete CNS repair responses, as mechanisms contributing to persistently high CNS viral loads 15 dpi, and age-dependent lethality. Additional kinetic analysis of CNS viral loads between 10 and 15 dpi may clarify the role of sustained POWV CNS levels as determinants of age-dependent lethality.

In all mice surviving POWV infection (30 dpi), we found POWV RNA levels near background while spongiform damage and reactive glial cells were persistent and pervasive in the CNS. Reactive glial cells were increased in all age mice 5–30 dpi suggesting that CNS repair and tissue remodeling are ongoing during early and acute stages of POWV infection as well as in survivors 30 dpi (68–70). Our findings suggest that persistent CNS POWV loads and reactive glial cell responses may contribute to age-dependent POWV lethality as well as long-term neurodegenerative damage or CNS inflammation in mice. This is consistent with 10% lethality and severe long-term neurologic sequelae observed in ~50% of surviving POWV patients (2, 7, 14, 17). It remains to be determined whether POWV-directed CNS pathology in mice causes long-term cognitive sequelae and whether cognitive changes are age independent.

Reactive glial cells are principal mediators of neuroinflammation and age-dependent neurodegenerative diseases (44, 58, 59, 63, 65, 67, 76, 91, 99). Microglia are resident CNS immune cells that mediate innate phagocytic responses and subsequent repair *via* distinct neuroprotective or neurotoxic responses (44, 58, 59, 62, 63, 68–70, 91). In a simplified view, microglia take on reactive pro-inflammatory M1 or, neuroprotective M2 microglial phenotypes, forming a continuum of intermediate types dependent on localized cell and cytokine stimuli (59, 91, 92). M1 microglia are activated by IFNγ and highly secrete proinflammatory Th1-type cytokines (TNFα, IFNγ, IL-1β, IL-2, IL-6, and IL-12) and immune cell recruiting chemokines (44, 58, 59, 63, 67, 76, 91, 99). By contrast, CCL2-activated M2 microglia secrete immunoregulatory (IL-10, TGFβ) and neuroprotective (IL-4, IL-13) Th2-type cytokines (51, 56, 57). Astrocytes also maintain CNS homeostasis, with reactive astrocytes inducing GFAP expression, activation of microglial RAGE receptors, and augmented microglial inflammation in the CNS (46–48, 56, 67, 83, 87, 100). As a result, altering the balance of neurotoxic and neuroprotective glial cells in the CNS may direct damaging inflammatory CNS responses and prevent POWV clearance from the CNS (44, 47, 59, 62, 63, 67, 80, 82–84, 91, 101).

Glial cell activation is reported in the CNS of TBEV-infected mice and humans (102, 103) as well as in encephalitis caused by TBEV, WNV, and Japanese encephalitis virus (JEV) (40, 79, 81, 95, 99, 104–106). The activation of glial cells in all POWV-infected mice suggests that divergent glial cell responses may distinguish age-dependent POWV clearance and survival from lethal outcomes (44, 80, 82, 84, 101, 107). Analysis of CNS cytokine responses in POWV-infected mice revealed the induction of immunosuppressive IL-10 and TGFβ (354-, 14-fold) in 10-week-old mice 15 dpi, and the induction of pro-inflammatory cytokines IL-2 and IL-12 (>2,800 fold), TNFα, IL-6, IFNγ, and IL-1β (230-, 66-, 154-, 10-fold) in the CNS of 50-week-old mice. IL-4 was induced in both 10- and 50-week-old mice but is accompanied by induced IL-2 and IL-12 cytokines in 50-week-old mice, which provide a proinflammatory IL-4 context (51, 56, 59, 87, 91, 108). The high induction of Th1 cytokines in the CNS suggests a neurodegenerative M1/Th1 microglia phenotype in LI9-infected 50-week-old mice, that may contribute to CNS damage, ineffective POWV clearance, and POWV lethality (51, 52, 55, 56, 59, 63, 87). By contrast, the induction of Th2-type cytokines in the CNS of 10-week-old mice reflects a neuroprotective, anti-inflammatory M2 microglial phenotype (57, 59–61, 63, 84, 87) that may support CNS repair, POWV clearance, and restrict lethality. Detailed analysis of CNS cells, cytokine responses, and their timing in relation to POWV CNS viral clearance is needed to discern causes of survival and lethality in young versus aged mice.

In murine WNV studies, kinetic CNS analysis and glial cell responses remain to be factored into age-dependent severity, with suggested increases in CD4/CD8 T cells and CD8 reactivation reported to distinguish WNV clearance in young versus aged mice (79, 93, 95, 99, 104–106). By contrast, we observed few CD4/CD8 T-cell infiltrates in the CNS, and no discernible differences in T-cell infiltrates in POWV-infected 10- versus 50-week-old mice (79, 95). Our findings associate glial cell activation with kinetic changes in CNS cytokine responses directing a neuroinflammatory state that results in age-dependent POWV lethality and long-term CNS pathology in survivors. It remains to be determined whether cytokine responses of young mice reveal potential therapeutic targets and approaches for preventing severe/lethal disease in aged mice, and POWV infections of the elderly.

Neuronal damage directs CNS tissue regeneration through neuronal stem cell (NSC) and microglial proliferation; however, reactive glial cells can also inhibit CNS regeneration (101, 109–111). Aging is known to decrease regenerative NSCs and increase senescent microglia and T cells (108, 112, 113) that persistently secrete pro-inflammatory cytokines (101, 110, 112, 114–116) causing an age-dependent senescence-associated secretory phenotype (SASP) (2, 13, 16, 117–119). In the CNS, the expression of IFNα, IL-2, and IL-12 is linked to experimental autoimmune encephalitis (EAE), multiple sclerosis (MS), Alzheimer’s disease (AD), and Parkinson’s disease (PD) (59, 84, 87, 117, 118, 120–122) through amplified Th1 responses (51, 52, 55, 56, 87, 123). In AD and PD, age-dependent cognitive deficits are also associated with the accumulation of senescent NSCs and microglia in a severe proinflammatory state (112, 114). CNS cytokine responses of POWV-infected 50-week-old mice reflect induced senescent cell cytokines (TNFα, IL6, and IL-1β) that may contribute to SASP. Anti-inflammatory steroids inhibit IL-12 and Th1 responses, and either blocking IL-12 or enhancing Th2 responses reduces AD pathology (87). There is a single report that five corticosteroid-treated POWV patients survived, while POWV was lethal in 5/5 untreated patients > 60 years of age (14). This anecdotal finding suggests the potential for steroids to suppress late neuroinflammatory POWV damage, and our lethal POWV murine model provides a means for assessing the efficacy of anti-inflammatory approaches. However, whether CNS repair, senescence or inflammatory glial cell responses contribute to age-dependent POWV lethality remains to be examined.

Our findings reveal a novel age-dependent murine model of lethal POWV encephalitis and long-term neurologic damage in survivors. Histopathology of POWV-infected aged murine brains mimics POWV-induced CNS pathology in fatal human cases (16) and long-term spongiform damage observed in survivors irrespective of age (2, 16). Our results associate Th2-type cytokine responses with POWV clearance and survival in young mice, and Th1-type cytokine responses and increased CNS viral loads with the lethality observed in 50-week-old mice. Our studies suggest roles for persistently activated glial cells, neurodegenerative M1/Th1 type cytokine responses, and failed POWV clearance with lethal outcomes in aged mice. This murine model provides a basis for defining the protective responses of young mice and age-dependent causes of POWV lethality. Further studies of young versus aged mice are likely to suggest therapeutic targets and approaches for resolving age-dependent POWV lethality in elderly patients and may be applicable to other age-dependent viral and chronic neurodegenerative diseases.

## MATERIALS AND METHODS

### Cells and virus

VeroE6 cells (ATCC CRL 1586) were grown in Dulbecco’s modified Eagle’s medium at 37°C in 5% CO_2_ as previously described (21, 124). POWV strain LI9 (GenBank accession number: MZ576219) was isolated from infected *Ixodes scapularis* ticks *via* inoculation on Vero E6 cells, and stocks passaged 3–4 in VeroE6 cells (21, 124). POWV LI9 was adsorbed onto 60% confluent VeroE6 monolayers for 1 h. Monolayers were PBS washed and grown in DMEM 5% FBS and POWV titers were determined by serial dilution and quantifying infected VeroE6 cell foci 1 dpi by immunoperoxidase staining with anti-POWV hyperimmune mouse ascites fluid (HMAF; 1:5,000; ATCC) (21, 42, 60). All work with infectious LI9 POWV was performed in a certified BSL3 suite.

### Biosafety and biosecurity

Animal research was performed in accordance with institutional guidelines using approved experimental protocols, and supervised by the Institutional Biosafety and Institutional Animal Care and Use Committees at SBU. Animals were managed by the SBU Division of Laboratory Animal Resources, which is accredited by the American Association for Accreditation of Laboratory Animal Care and DHHS, and maintained in accordance with the Animal Welfare Act and DHHS “Guide for the Care and Use of Laboratory Animals.” Veterinary care was directed by full-time resident veterinarians accredited by the American College of Laboratory Animal Medicine. POWV murine infection experiments were performed in an animal biosafety level 3 facility (The Laboratory of Comparative Medicine, Stony Brook University).

### Murine inoculation

C57BL/6J mice (10–50 weeks old) were purchased from Jackson Laboratory. Mice were anesthetized *via* intraperitoneal injection with 100 mg of ketamine and 20 mg of xylazine per kilogram of body weight. Animals were infected by subcutaneous footpad injection with up to 2 × 10^3^ FFU POWV or PBS in a volume of 20 µL. Mice were weighed daily and evaluated for signs of clinical neurologic disease. Mice that reached humane endpoints including non-responsiveness or severe neurologic signs (hindlimb flaccid paralysis, ataxia, inability to self-right) were euthanized from 1 to 20 dpi or at predetermined 5, 10, 15, and 30 dpi time points (64). Murine POWV inoculation and lethality analysis were replicated in at least three independent experiments.

### Histopathology

Brains were harvested postmortem, fixed in neutral buffered formalin for 7 days, dehydrated with 70% ethanol for 24 h, and paraffin embedded. Formalin-fixed paraffin-embedded (FFPE) brain tissues were sectioned (10 µm thickness) and hematoxylin and eosin (H&E) stained by the SBU Histology Core Lab. Immunohistochemical staining was performed by HistoWiz, Inc. with anti-Iba1 antibody (Wako 019–19741), anti-GFAP (NB300-141), anti-CD4 (ab183685), anti-CD8 alpha (CST98941), or anti-NeuN (ab177487) antibodies to identify microglia/macrophage, astrocytes, CD4 T cells, CD8 T cells, and neurons, respectively. ImageJ was used to quantify Iba1^+^ and GFAP^+^ immunostaining: the relative percent area of Iba1^+^ or GFAP^+^ pixel intensity was determined for 10 CNS regions of 10- and 50-week-old mice (*n* = 4), 5, 10, and 15 dpi, compared to age-matched mock-infected controls. Stained tissue sections were analyzed using QuPath software (https://qupath.github.io).

Histopathology scoring of H&E stained age-matched brain tissues was performed blinded to age and experimental group. Brain regions were scored on a scale of 0–4 for spongiform encephalopathy, microgliosis, and neuronal necrosis. Scores define localized severity of pathology: (0) baseline of age-matched control brain staining in select region, (1) localized lesion, (2) multiple localized lesions, (3) lesions spread throughout most of selected region, and (4) lesions uniformly spread throughout select region (71). ImageJ quantification of Iba1 immunostaining of 50-week-old mouse brains versus age-matched mock- infected controls: relative percent area of Iba1^+^ pixel intensity for 10 regions was determined per POWV-infected brain and compared to mock-infected controls. Brain section quantitation is derived from ImageJ analysis of at least 10 brain locations from each of four individual mice.

For RNA *in situ* hybridization (ISH), FFPE brain tissues were sectioned (5 µm thickness), xylene deparaffinized, and antigen retrieval treated in a decloaking chamber for 1 h. POWV RNA was detected using the RNAScope Universal AP assay (Advanced Cell Diagnostics, Inc.) according to the manufacturer’s protocol. RNAScope 2.3 VS probe V-Powassan (catalog #415641) was used to detect genomic POWV RNA (27). ISH-probed slides were analyzed using QuPath software.

### RNA extraction and qRT-PCR analyses

Age-matched mock or POWV-infected mice were euthanized and brains were harvested in TRIzol LS Reagent (Invitrogen) homogenized and RNA purified according to the manufacturer’s protocols. RNA was processed using Monarch RNA Cleanup Kit (NEB T2030L) and quantified on a Nanodrop Spectrophotometer 2000. To define viral loads and inflammatory transcripts in POWV-infected brains, qRT-PCR was performed on purified RNAs from brains of age-matched mock and POWV-infected mice (21, 42, 115). cDNA synthesis was performed using random hexamer priming of a Transcriptor first-strand cDNA synthesis kit (Roche) as previously described (21, 42, 60). Viral RNA levels were assayed using NS5-specific LI9 primers (Table 2) and compared to a standard curve of serially diluted POWV RNA. qRT-PCR transcript primers were designed using the NCBI gene database, with 60°C annealing profiles (Table 2). Transcript levels were analyzed in triplicate from *n* = 3 age-matched control or *n* = 3 POWV-infected mice using PerfeCTa SYBR green SuperMix (Quanta Biosciences) on a Bio-Rad C1000 Touch system with a CFX96 optical module (Bio-Rad). Responses were normalized to internal GAPDH mRNA levels, and the fold induction was calculated using the threshold cycle (2^-ΔΔCT^) method for differences between age-matched mock and POWV-infected RNA levels at each time point post-infection.

**TABLE 2:** Primers used in qRT-PCR analysis

Gene		
GAPDH	Forward primer	AATGGTGAAGGTCGGTGTG
	Reverse primer	GTGGAGTCATACTGGAACATGTAG
CXCL10	Forward primer	AGTGCTGCCGTCATTTTCTG
	Reverse primer	ATTCTCACTGGCCCGTCAT
CCL2	Forward primer	TCAGCCAGATGCAGTTAACG
	Reverse primer	CTCTCTTGAGCTTGGTGACA
CCL5	Forward primer	CAAGTGCTCCAATCTTGCAG
	Reverse primer	CCTCTATCCTAGCTCATCTCCA
IFNλ	Forward primer	TGCAACCAATCATGCCGTGT
	Reverse primer	CCCATTCATCGCTAGGTGGTC
IFNβ	Forward primer	CTGGAGCAGCTGAATGGAAAG
	Reverse primer	CTTCTCCGTCATCTCCATAGGG
IL-10	Forward primer	CCCTTTGCTATGGTGTCCTT
	Reverse primer	TGGTTTCTCTTCCCAAGACC
IL-1β	Forward primer	GGAGAACCAAGCAACGACAAAATA
	Reverse primer	TGGGGAACTCTGCAGACTCAAAC
IL-2	Forward primer	AACCTGAAACTCCCCAGGAT
	Reverse primer	CGCAGAGGTCCAAGTTCATC
IL-4	Forward primer	GAATGTACCAGGAGCCATATC
	Reverse primer	CTCAGTACTACGAGTAATCCA
IL-6	Forward primer	TGGGAAATCGTGGAAATGAG
	Reverse primer	CTCTGAAGGACTCTGGCTTTG
IL-7	Forward primer	CTTGTTCTGCTGCCTGTCAC
	Reverse primer	CTTGCGAGCAGCACGATTTAG
IL-12	Forward primer	GGAAGCACGGCAGCAGAATC
	Reverse primer	AACTTGAGGGAGAAGTAGGAATGG
IL-13	Forward primer	AGACCAGACTCCCCTGTGCA
	Reverse primer	TGGGTCCTGTAGATGGCATTG
IL-15	Forward primer	CATCCATCTCGTGCTACTTGTGTT
	Reverse primer	CATTATCCAGTTGGCCTCTGTTT
IL-18	Forward primer	CAGGCCTGACATCTTCTGCAA
	Reverse primer	TCTGACATGGCAGCCATTGT
TNF	Forward primer	CGTCGTAGCAAACCACCAAG
	Reverse primer	TTGAAGAGAACCTGGGAGTAGACA
TGF-β	Forward primer	CAGTGGCTGAACCAAGGAGAC
	Reverse primer	ATCCCGTTGATTTCCACGTG
ARG-1	Forward primer	ATGGAAGAGACCTTCAGCTAC
	Reverse primer	GCTGTCTTCCCAAGAGTTGGG
SOCS3	Forward primer	ACCTTCAGCTCCAAAAGCGAGTAC
	Reverse primer	CGCTCCAGTAGAATCCGCTCTC
POWV NS5	Forward primer	GAAACAATACTCAGAATCATG
	Reverse primer	AAGCCGCTGATCCAGTGGCA

### Neutralizing antibody assays

C57BL/6 10- to 50-week-old mice were s.c. footpad inoculated with 2 × 10^3^ FFU of POWV LI9 or mock infected with PBS. Neutralizing antibodies present in sera from POWV LI9, or mock-infected mice were assessed at 30 dpi using a POWV LI9 focus reduction neutralization assay. Sera were serially diluted and added to 500 FFU of POWV LI9 for 1 h prior to adsorption to VeroE6 cells, and 36 hpi cells were immunoperoxidase stained using anti-POWV hyperimmune mouse ascites fluid (HMAF; 1:5,000; ATCC), horseradish peroxidase (HRP)-labeled anti-mouse IgG (1:2,000; KPL-074–1806), and 3-amino-9-ethylcarbazole (AEC) (21, 42, 115). POWV-infected cells were quantitated and serum dilutions required to reduce POWV foci by 50% (IC50s) were calculated and presented as a range of neutralizing antibody responses (*n* = 4/age group).

### Statistical analysis

The statistical significance of the results was determined using Prism 6 software (GraphPad Software, Inc.; https://www.graphpad.com). Statistical analysis for individual experiments is presented in the figure legends. The infection of young vs aged mice has been replicated in at least three independent experiments and additionally by determining the minimal POWV dose required for lethality. Histopathology analyses are compared across groups using one or two-way ANOVA with replicate sample sizes having *n* = 4 mice/age and time points in all figures (71). The number of animals for the histopathology study has been calculated based on the minimal number needed to achieve statistical significance. Based on prior studies, the values obtained for the mean and standard deviation for the control and experimental groups suggest that four animals per group would provide 80% power (Alpha of 0.05) to detect a change of 2 in histological scores. Kaplan-Meier curves were analyzed by a log rank test. *P* values of less than 0.05 were considered statistically significant.

## SUPPLEMENTAL MATERIAL

Fig. S1 jvi.00560-24-s0001.tiff Kinetic changes in the pons of POWV infected 20-, 30-, and 40-week-old mice.

Fig. S2 jvi.00560-24-s0002.tiff POWV causes neuronal depletion without disrupting Purkinje cells.

Fig. S3 jvi.00560-24-s0003.tiff POWV causes spongiform encephalitis, microgliosis, and neuronal necrosis in mice of all ages.

Fig. S4 jvi.00560-24-s0004.tiff POWV-infected CNS in situ hybridization.

Fig. S5 jvi.00560-24-s0005.tiff POWV-induced responses of 50- vs 10-week-old mice, 15 dpi.

Supplemental legends jvi.00560-24-s0006.docx Legends for Fig. S1 to S5.

ASM does not own the copyrights to Supplemental Material that may be linked to, or accessed through, an article. The authors have granted ASM a non-exclusive, world-wide license to publish the Supplemental Material files. Please contact the corresponding author directly for reuse.
