# Supplementary figures and images for: Age-dependent Powassan virus lethality is linked to glial cell activation and divergent neuroinflammatory cytokine responses in a murine model
Source: J Virol. 2024 Aug 1;98(8):e00560-24. doi: 10.1128/jvi.00560-24 (PMC11334436; doi:10.1128/jvi.00560-24)

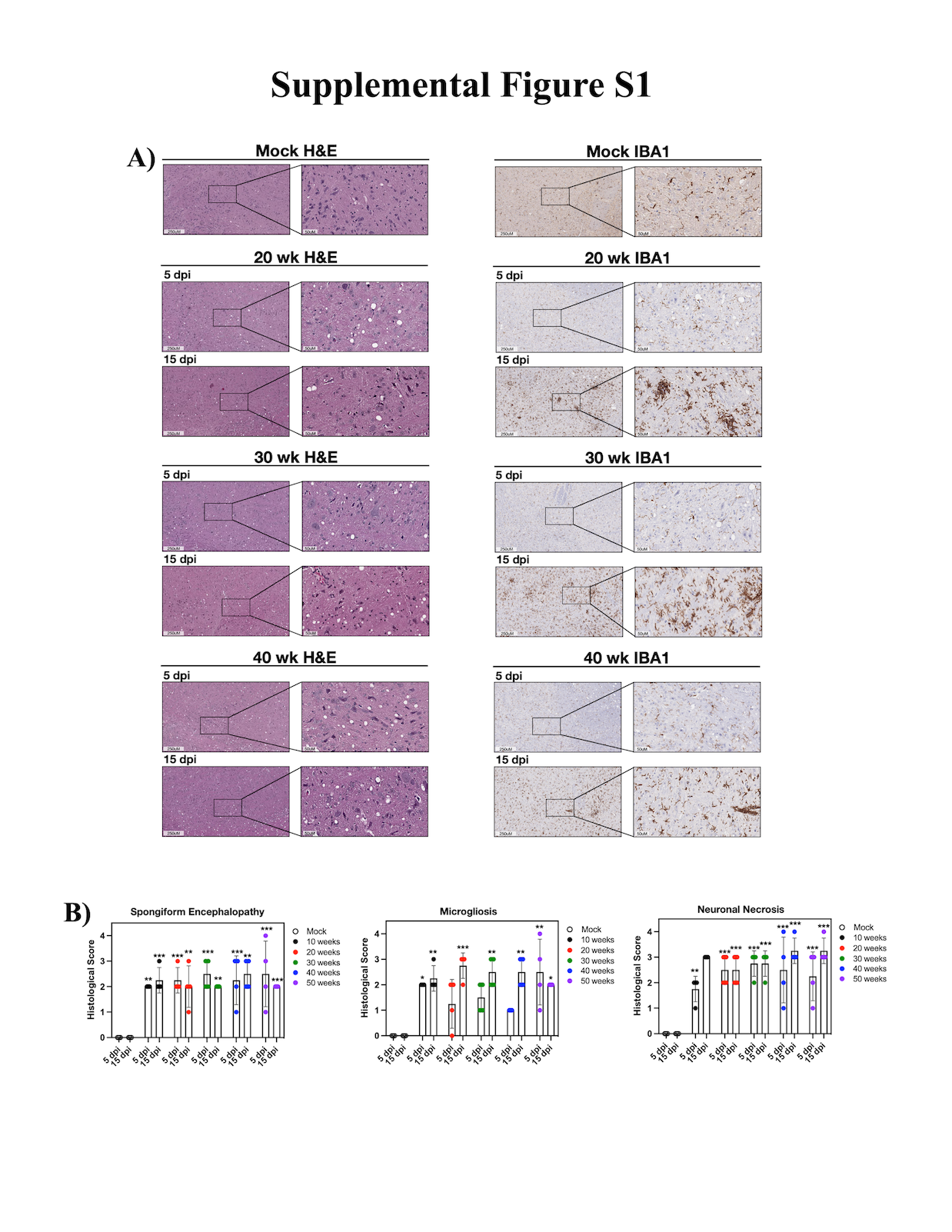

Supplement: Fig. S1 — Kinetic changes in the pons of POWV infected 20-, 30-, and 40-week-old mice. [file jvi.00560-24-s0001.tiff]

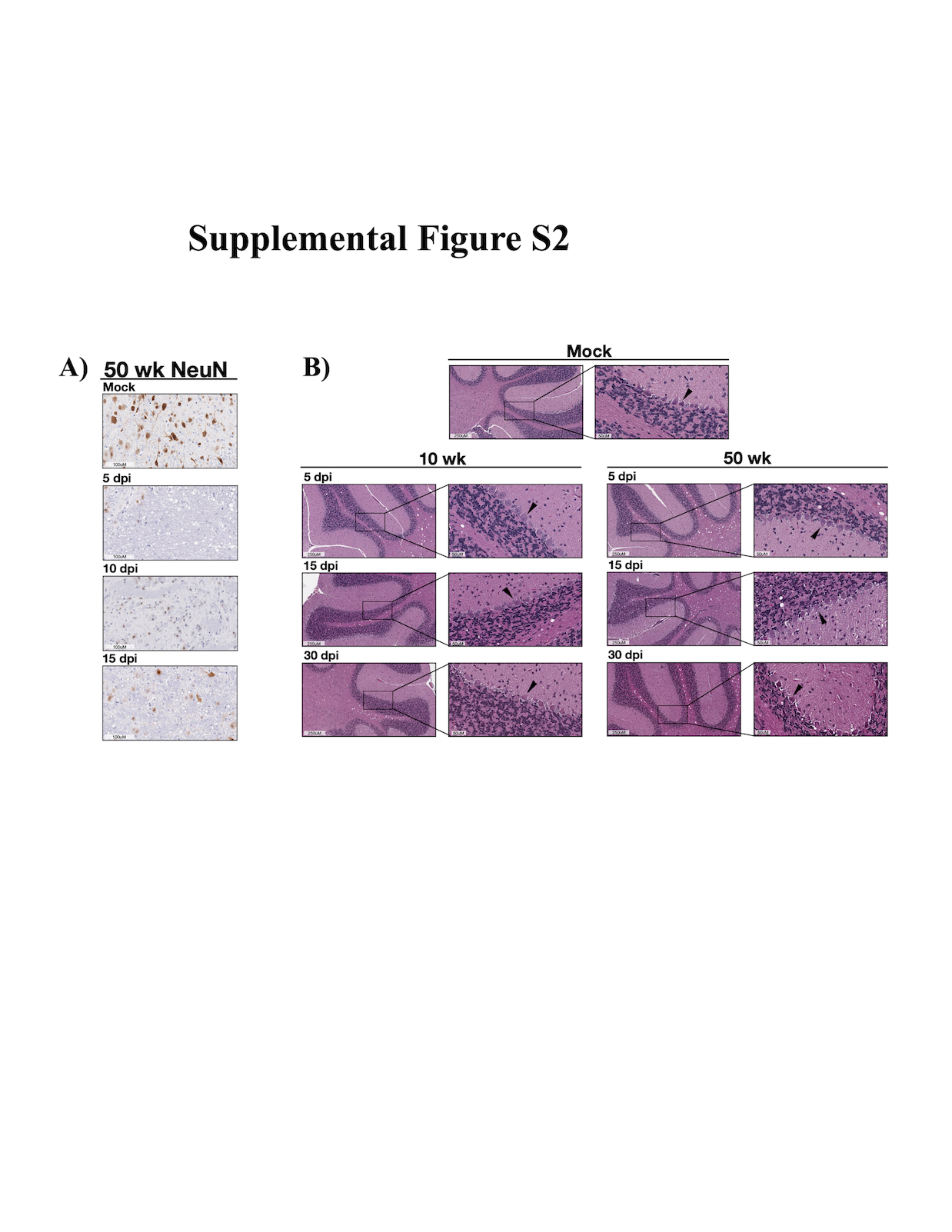

Supplement: Fig. S2 — POWV causes neuronal depletion without disrupting Purkinje cells. [file jvi.00560-24-s0002.tiff]

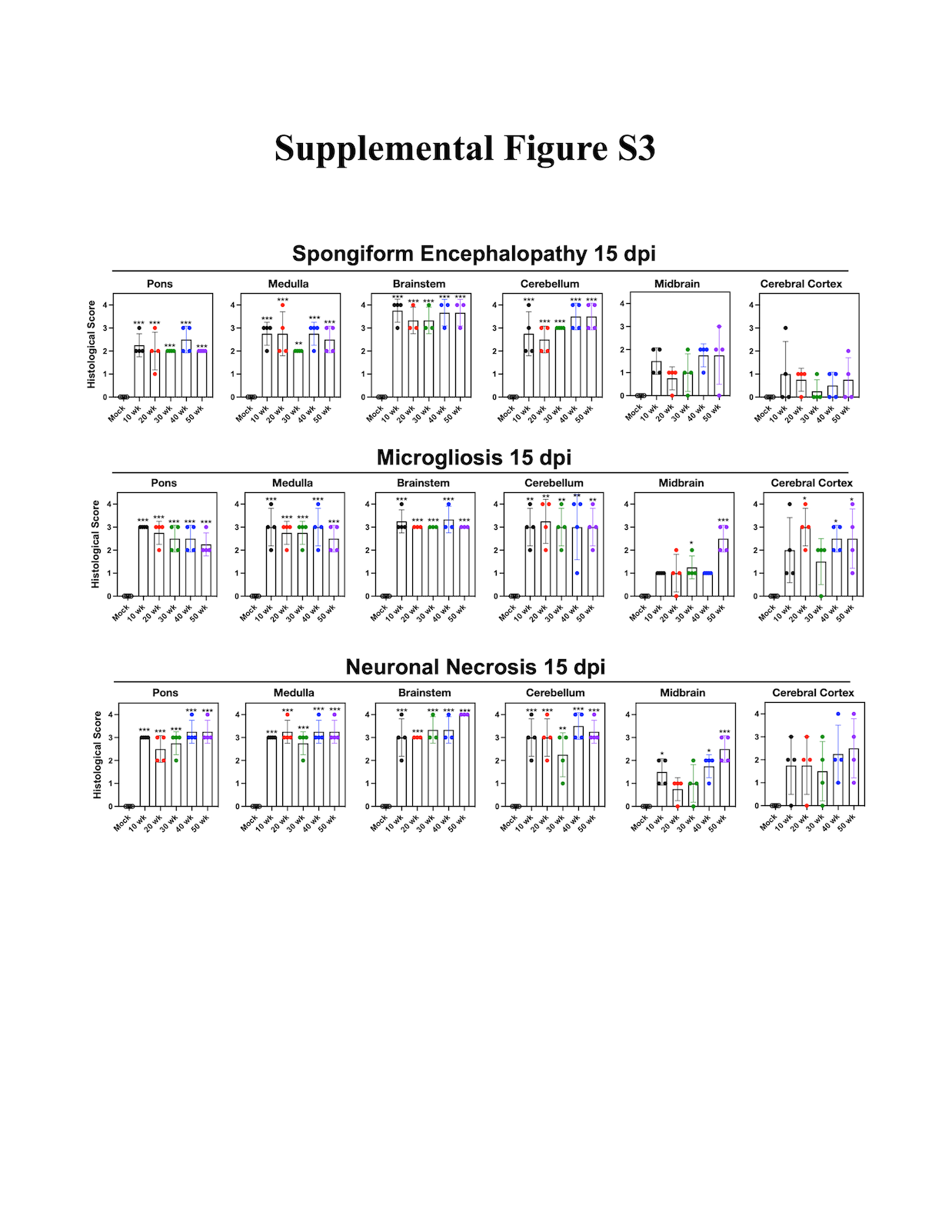

Supplement: Fig. S3 — POWV causes spongiform encephalitis, microgliosis, and neuronal necrosis in mice of all ages. [file jvi.00560-24-s0003.tiff]

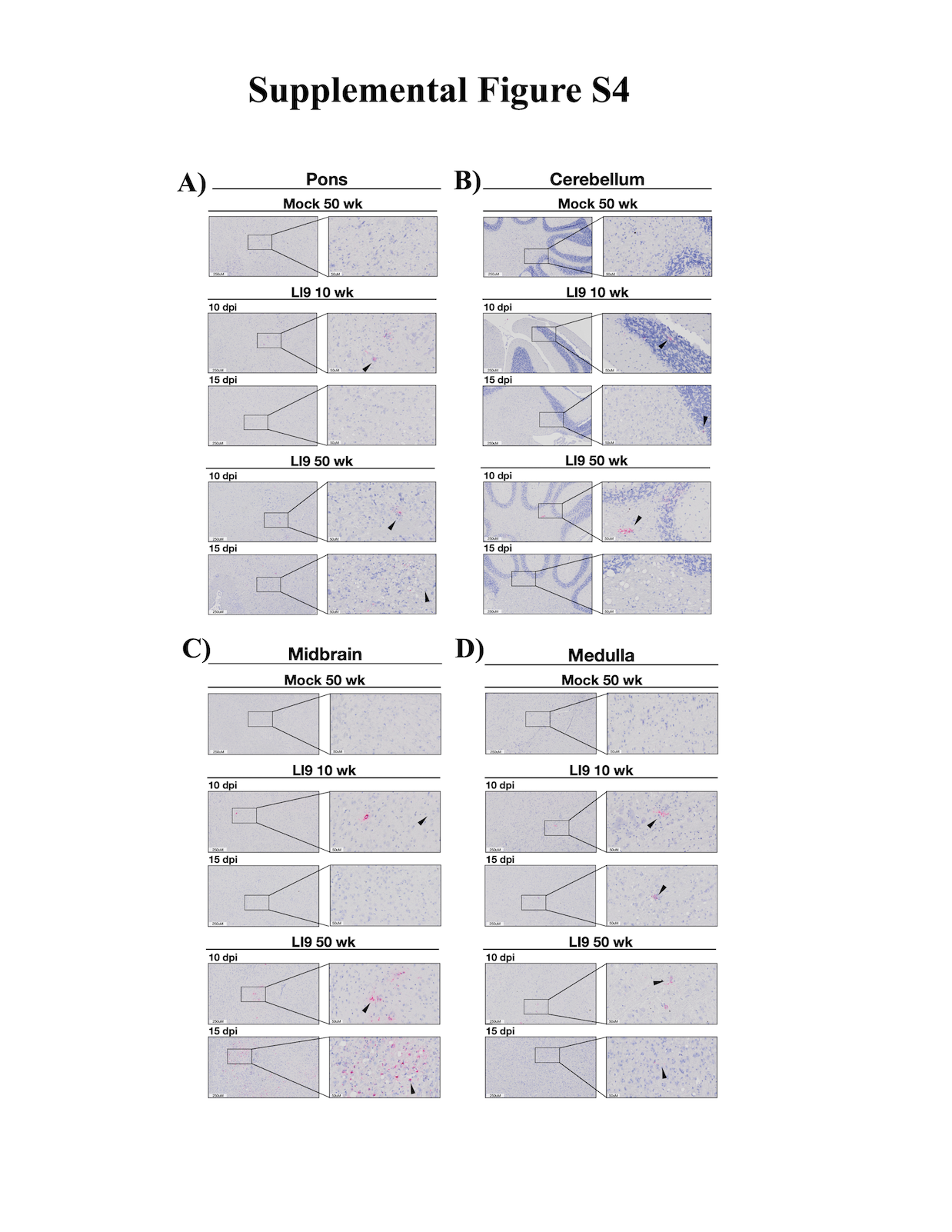

Supplement: Fig. S4 — POWV-infected CNS in situ hybridization. [file jvi.00560-24-s0004.tiff]

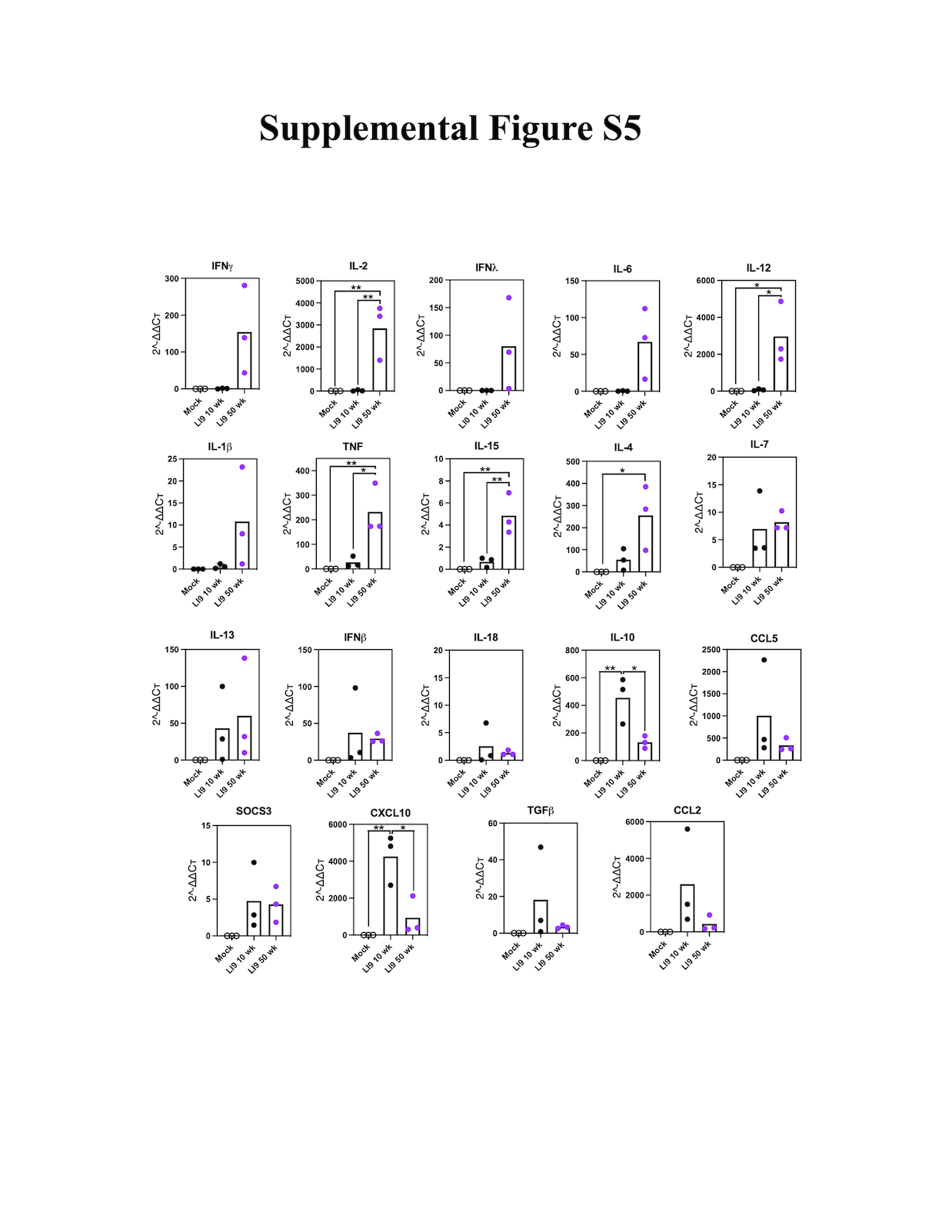

Supplement: Fig. S5 — POWV-induced responses of 50- vs 10-week-old mice, 15 dpi. [file jvi.00560-24-s0005.tiff]
