# Supplementary material for: Age-dependent Powassan virus lethality is linked to glial cell activation and divergent neuroinflammatory cytokine responses in a murine model
Source: J Virol. 2024 Aug 1;98(8):e00560-24. doi: 10.1128/jvi.00560-24 (PMC11334436; doi:10.1128/jvi.00560-24)
Supplement: Supplemental legends — Legends for Fig. S1 to S5. [file jvi.00560-24-s0006.docx]

**SUPPLEMENTAL FIGURES**

**Figure S1. Kinetic Changes in the Pons of POWV Infected 20, 30 and 40 Week Old Mice. (A)** C57BL/6 mice 20, 30 and 40 weeks of age were footpad inoculated with 2 x 10^3^ FFU of POWV LI9 or mock infected with PBS. Brains were harvested 5 and 15 dpi, and H&E stained (n=4), or immunostained for microglia/macrophages (n=4) using anti-Iba1 antibody. Representative sections of the Pons are presented. **(B)** The severity of POWV directed spongiform encephalopathy, microgliosis, and neuronal necrosis in H&E stained Pons regions (n=4) were scored on a scale of 0-4 by blinded comparison versus age-matched controls: (0) baseline determined by control brain staining in select region, (1) localized lesion, (2) multiple localized lesions, (3) lesions spread throughout most of select region, (4) lesions uniformly spread throughout select region^(71)^. Data are presented as means with standard deviation (SD) and each dot represents an individual mouse. Individual data point comparisons were performed by two-way ANOVA analysis. Asterisks indicate statistical significance (*, *P<*0.01; **, *P<*0.001, ***, *P<*0.0001).

**Figure S2. POWV Causes Neuronal Depletion Without Disrupting Purkinje Cells.**

**(A-B)** C57BL/6 10 and 50 week old mice were footpad inoculated with 2 x 10^3^ FFU of POWV LI9 or mock infected with PBS. **(A)** Brains were harvested 5, 10 and 15 dpi, and immunostained for neurons (n=4) using NeuN antibody. Representative sections of the Pons are presented. **(B)** Brains were harvested 5, 15, or 30 dpi, sectioned, and H&E stained. Representative images of the cerebellum in POWV LI9 infected and mock infected 50 week old mice are presented. Black arrows indicate representative Purkinje Cells.

**Figure S3. POWV Causes Spongiform Encephalitis, Microgliosis and Neuronal Necrosis in Mice of All Ages.**  C57BL/6 mice 10-50 week old were footpad inoculated with 2 x 10^3^ FFU of POWV LI9 or mock infected with PBS. Brains were harvested 15 dpi, sectioned and H&E stained (n=4). Representative images of CNS histopathology in the Pons, Medulla, Brainstem, Cerebellum, Midbrain and Cerebral cortex of POWV versus mock infected 50 week old brains 15 dpi were scored for spongiform encephalopathy, microgliosis, and neuronal necrosis (n=4) as in Figure S1B. Data are presented as means with SD and each dot represents an individual mouse. Individual data point comparisons were performed by one-way ANOVA analysis. Asterisks indicate statistical significance (*, *P<*0.01; **, *P<*0.001, ***, *P<*0.0001).

**Figure S4. POWV Infected CNS In Situ Hybridization.**  C57BL/6 10-50 week old mice were footpad inoculated with 2 x 10^3^ FFU of POWV LI9 or PBS. Brains from POWV infected or mock-infected 10 and 50 week old mice were harvested 15 dpi, and genomic POWV RNA in the CNS was detected by in situ hybridization (ISH) with POWV RNAscope probe-red. **(A)** Pons; **(B)** Cerebellum; **(C)** Midbrain; **(D)** Medulla. Representative images of POWV RNA ISH in POWV infected (n=4) or mock infected 50 week old mice (n=1) are presented. Black arrows indicate representative cells positive for genomic POWV RNA by ISH.

**Figure S5. POWV Induced Responses of 50 vs 10 Week Old Mice, 15 dpi.** C57BL/6 10 and 50 week old mice were footpad inoculated with 2 x 10^3^ FFU of POWV LI9 or mock infected with PBS. Brains from POWV or mock infected mice were harvested, total RNA extracted and the induction of cytokine and chemokine transcripts were in the CNS were assayed by qRT-PCR, standardized to GAPDH RNA levels and compared to mock infected age-matched controls. Each time point, age group and infected or mock infected age-matched control is presented (n=3). Data are presented as means and each dot represents an individual mouse. Individual data point comparisons were performed by one-way ANOVA analysis. Asterisks indicate statistical significance (**p<*0.05; ***p<*0.01)
